# A negative feedback cascade controlling jasmonate-induced nicotine biosynthesis revealed by an in-plate nicotine assay

**DOI:** 10.1186/s43897-026-00249-4

**Published:** 2026-09-08

**Authors:** Qing Xu, Ke Xu, Haihui Cao, Yulong Gao, Peng Li, Wenjing Wang, Yong Chen, Michael P. Timko, Zhongfeng Zhang, Bingwu Wang, Hongbo Zhang

**Affiliations:** 1 Key Laboratory of Synthetic Biology of Ministry of Agriculture and Rural Affairs, Tobacco Research Institute, Chinese Academy of Agricultural Sciences, Qingdao, 266101 China; 2 Yunnan Academy of Tobacco Agricultural Sciences, Kunming, 650021 China; 3 Beijing Life Science Academy (BLSA), Beijing, 102209 China; 4 Department of Biology, University of Virginia, Charlottesville, VA 22904 USA

Nicotine is the principal alkaloid in tobacco that confers defense against herbivores (Wari et al. 2021; Shoji et al. 2024). Activation of nicotine biosynthesis is mediated by jasmonate (JA) signaling transcription factor NtMYC2s that specifically bind to G-boxes in the promoters of nicotine biosynthetic genes (Zhang et al. 2012; Sui et al. 2021; Hou et al. 2023). While prior researches focused on their positive functions, this study reports a negative role of NtMYC2a in regulating JA-induced nicotine biosynthesis.

Recently, we observed that topping-induced nicotine increase in *NtMYC2a*-overexpressing tobacco (*NtMYC2a*-OE) was not higher than that in control (Bian et al. 2022; Fig. S1) and suspected the involvement of NtMYC2a in a negative feedback mechanism that prevents excessive nicotine accumulation. In order to investigate the underlying mechanism, an in-plate nicotine production assay (IPNPA) was developed to evaluate the nicotine biosynthesis capacity of tobacco plants. The IPNPA allows for rapid and reliable evaluation of nicotine biosynthesis in tobacco using plantlets cultured on solid MS (Murashige & Skoog) medium supplemented with MeJA, a methyl ester of JA that mediates topping responses and nicotine biosynthesis in tobacco. Results showed that wild type tobacco grown on MS medium accumulated 11.7 µg/mg nicotine with 3 µM MeJA supplementation and 18.1 µg/mg nicotine with 5 µM MeJA supplementation (Fig. 1a), which is comparable to field-grown tobacco (Hou et al. 2023; Shi et al. 2025). Supplementation of 3 µM MeJA was selected for subsequent assays, for it was sufficient to activate nicotine biosynthesis and caused less growth inhibition on tobacco plantlets than 5 µM MeJA (Fig. 1a). The IPNPA was further validated using a series of low-nicotine *nic* mutants, which showed a reliability in evaluating plants with different nicotine production (Fig. S2), and was then employed to analyze the nicotine production in *NtMYC2a*-OE plants. As shown in Fig. 1b, the nicotine content of *NtMYC2a*-OE plants was 5.2 µg/mg in the absence of MeJA and 12.6 µg/mg in the presence of MeJA, while that of control was 3.1 µg/mg without MeJA treatment and 11.0 µg/mg with MeJA treatment. Thus, the JA-induced nicotine increase in *NtMYC2a*-OE plants was approximately 140%, significantly lower than the 260% increase in control plants (Fig. 1b). Moreover, *NtMYC2a*-RI plants had much lower nicotine content, but remained similar extent of JA-induced nicotine increase as control (Fig. 1b). These results evidence that *NtMYC2a* overexpression restricts the JA-induced nicotine production. Subsequent qRT-PCR analysis revealed that the JA-induced expression of *NtODC* and *NtADC* (encoding ornithine decarboxylase and arginine decarboxylase, respectively) was greatly suppressed in *NtMYC2a*-OE plants (Fig. 1c), while that of *NtPMT*, *NtQPT*, *NtA622*, and *NtBBL* was higher than in control (Fig. S3).

**Fig. 1:**
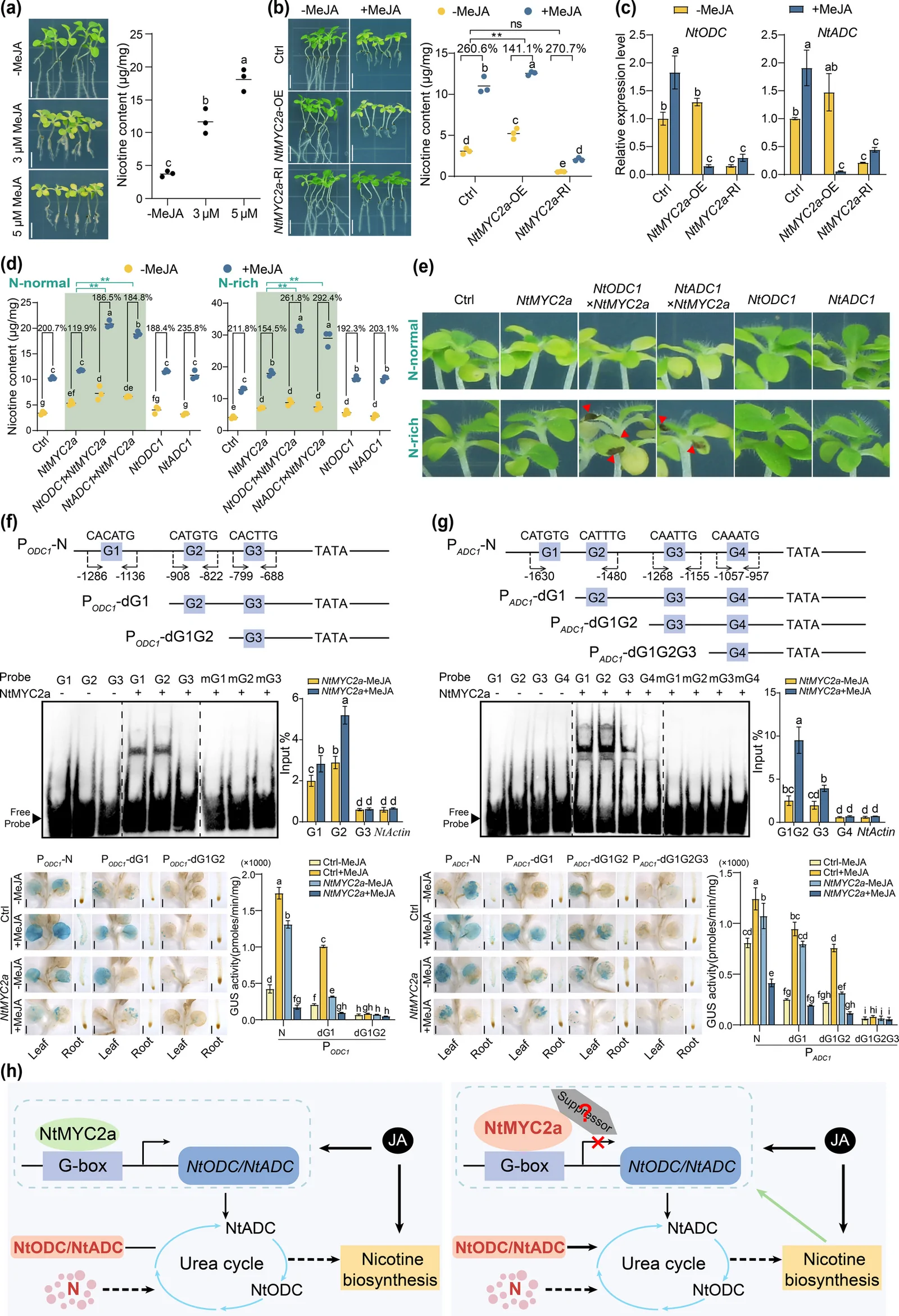
NtMYC2a-mediated feedback regulation of JA-induced nicotine biosynthesis. **a** and **b** Phenotype and nicotine content of plants under indicated treatments. Wild type TN90 are used in (**a**). JA-induced nicotine increase in (**b**) is indicated. Ctrl, empty vector transformed. Bar = 0.5 cm. **c** Relative expression of *NtODC* and *NtADC*. Expression in untreated Ctrl was set as 1. **d** Nicotine content in plants under N-normal and N-rich conditions. JA-induced nicotine increase is indicated. **e** Representative phenotype of plants under N-normal and N-rich conditions with MeJA. Necrotic black spots are marked with red triangles. Bar = 0.5 cm. **f** and **g** NtMYC2a-binding regions in *NtODC1* (**f**) and *NtADC1* (**g**) promoters. Top panels illustrate promoter structures, with G-box sequences, ChIP–qPCR primer location and GUS assay truncation indicated. Prefix ‘m’ indicates mutant probes for EMSA and *NtActin* was used as internal control for ChIP–qPCR in middle panel. Bar = 2 mm in GUS staining (bottom panel). − MeJA, untreated; + MeJA, MeJA-treated. Asterisks (Student’s *t*-test, *P* < 0.05) and lowercase letters (Tukey’s test, *P* < 0.05) indicate significant differences. **h** Regulatory model of the NtMYC2a–*NtODC*/*NtADC* cascade in JA-mediated nicotine biosynthesis in the absence (left) or presence (right) of overexpressed *NtMYC2a*. Overexpressed *NtMYC2a* and *NtODC*/*NtADC* as well as N supply are indicated by red characters

To dissect the underlying regulation, *NtMYC2a*-OE plants with *NtODC1*/*NtADC1* co-expression were generated by crossing *NtMYC2a*-OE plants with those expressing *NtODC1* (*NtODC1*-OE) or *NtADC1* (*NtADC1*-OE). The nicotine content of *NtODC1/NtADC1* co-expressing *NtMYC2a*-OE plants was ~7.0 µg/mg in the absence of MeJA and ~20.0 µg/mg in the presence of MeJA, while that of *NtMYC2a*-OE plants was 5.3 µg/mg in the absence of MeJA and 11.8 µg/mg in the presence of MeJA (Fig. 1d). Thus, the JA-induced nicotine increase in *NtODC1/NtADC1* co-expressing *NtMYC2a*-OE plants was 185%, which was significantly higher than the 120% increase in *NtMYC2a*-OE plants and similar to that in control or *NtODC1/NtADC1*-OE plants (Fig. 1d). These results demonstrate that *NtODC1/NtADC1* co-expression boosts the JA-induced nicotine production in *NtMYC2a*-OE plants. Consistently, the JA-induced expression of nicotine biosynthetic genes was notably upregulated in *NtODC1/NtADC1* co-expressing *NtMYC2a*-OE plants (Fig. S4).

For NtADC and NtODC control the nitrogen metabolic flow to nicotine (Wang et al. 2008; Fig. S5), we investigated the effect of increased nitrogen supply on JA-induced nicotine production in *NtMYC2a*-OE plants. Under N-rich condition, the nicotine content of *NtMYC2a*-OE plants was 7.1 µg/mg in the absence of MeJA and 18.0 µg/mg in the presence of MeJA, and the JA-induced nicotine increase was 154.5% (Fig. 1d). The nicotine content of *NtODC1/NtADC1* co-expressing *NtMYC2a*-OE plants was ~8.0 µg/mg in the absence of MeJA and ~30.0 µg/mg in the presence of MeJA, and the JA-induced nicotine increase was over 260.0% (Fig. 1d). This increase is much higher than that in *NtMYC2a*-OE plants and even higher than that in control or *NtODC1/NtADC1*-OE plants (Fig. 1d). These findings indicate that *NtODC1/NtADC1*-expressing and nitrogen supply act synergistically in promoting the JA-induced nicotine increase in *NtMYC2a*-OE plants, providing evidence for the involvement of nitrogen metabolic flow in this process.

Nevertheless, necrotic black spots formed on the MeJA-treated *NtODC1/NtADC1* co-expressing *NtMYC2a*-OE plants under N-rich conditions (Fig. 1e, Fig. S6). This symptom might result from an autotoxic effect of overproduced nicotine, as a similar phenomenon was observed in *Epipremnum aureum* leaves uptaking excessive nicotine (Weidner et al. 2005). Accordingly, the fold change of nitrogen-enhanced expression of several nicotine biosynthetic genes was remarkably reduced in *NtODC1/NtADC1* co-expressing *NtMYC2a*-OE plants (Fig. S7). This should be a feedback reaction of tobacco plants in controlling nicotine overproduction, which may cause harm.

To determine how NtMYC2a regulate *NtODC1*/*NtADC1* expression, NtMYC2a-binding elements in their promoters were analyzed by bioinformatics, which identified three G-boxes in *NtODC1* promoter (designated as P_*ODC1*_-G1/G2/G3) and four G-boxes in *NtADC1* promoter (designated as P_*ADC1*_-G1/G2/G3/G4) as indicated in Fig. 1f, g. Similar G-boxes were also identified in the promoters of *NtODC1* and *NtADC1* homologs (Fig. S8). Electrophoretic Mobility Shift Assay (EMSA) using His-tagged NtMYC2a and synthesized DNA fragments as indicated showed clear mobility shifts in the reactions containing P_*ODC1*_-G1/G2 and P_*ADC1*_-G1/G2/G3, but no shift in that with P_*ODC1*_-G3, P_*ADC1*_-G4, or mutated probe (Fig. 1f, g). In vivo binding of NtMYC2a to *NtODC1*/*NtADC1* promoters was examined by Chromatin Immunoprecipitation (ChIP) assay using tobacco plants expressing HA-tagged NtMYC2a and an anti-HA antibody. The enrichment ratios for ChIP-enriched P_*ODC1*_-G1 and P_*ODC1*_-G2 were 2.0% and 2.9% in mock condition and were increased to 2.8% and 5.2% in the presence of MeJA, respectively (Fig. 1f, g). The enrichment ratios for P_*ADC1*_-G1G2 and P_*ADC1*_-G3 were 2.5% and 2.0% in mock condition and were increased to 9.5% and 3.9% in the presence of MeJA, respectively (Fig. 1f, g). On the contrary, the enrichment ratios for P_*ODC1*_-G3, P_*ADC1*_-G4 as well as *NtActin* were about 0.6% under both conditions (Fig. 1f, g). These results show that NtMYC2a predominantly binds to G1/G2 of *NtODC1* promoter and to G1/G2/G3 of *NtADC1* promoter*,* whose in vivo interaction was accentuated by MeJA treatment.

In transactivation assay, GUS expression driven by P_*ODC1*_-N as well as P_*ADC1*_-N/dG1 was observed in the shoots and roots of both control and *NtMYC2a*-OE plants, and showed stronger staining and notably higher GUS activity in *NtMYC2a*-OE plants (Fig. 1f, g). In contrast, GUS expression driven by P_*ODC1*_-dG1 and P_*ADC1*_-dG1G2 was less upregulated in *NtMYC2a*-OE plants, and that driven by P_*ODC1*_-dG1G2 or P_*ADC1*_-dG1G2G3 was almost undetectable in either genotype (Fig. 1f, g). These findings suggest that NtMYC2a mainly binds to P_*ODC1*_-G1 and P_*ADC1*_-G1/G2 to activate *NtODC1*/*NtADC1* expression in the absence of JA. Interestingly, MeJA treatment enhanced the GUS expression driven by P_*ODC1*_-N/dG1 as well as P_*ADC1*_-N/dG1/dG1G2 in control plants but suppressed the expression in *NtMYC2a*-OE plants with the same reporters (Fig. 1f, g). These results indicate that NtMYC2a binds to specific G-boxes in *NtODC1*/*NtADC1* promoters to regulate their transcription and that this regulation is attenuated by MeJA treatment in *NtMYC2a*-OE plants, which are consistent with above physiological results.

Using the IPNPA, which allows convenient and reliable evaluation of nicotine production in tobacco, this study unravels the role of NtMYC2a-mediated *NtADC/NtODC* expression in braking JA-induced nicotine biosynthesis and reveals a synergistic action of *NtADC1/NtODC1* co-expression and increased nitrogen supply in modulating this suppression. Based on currently observed functions of NtMYC2a in coordinating *NtADC1/NtODC1* expression and nitrogen metabolism for nicotine biosynthesis, we presume that unidentified suppressors may be involved in mediating the JA-induced braking of NtMYC2a-regulated nicotine biosynthesis (Fig. 1h). Taken together, findings of this work reveal a previously unknown function of the NtMYC2a-*NtADC/NtODC* cascade in regulating nicotine biosynthesis and establish the molecular interaction between NtMYC2a and *NtADC1*/*NtODC1* promoters.

## Supplementary Information

Supplementary Material 1: SI-1 Supplementary materials and methods. SI-2 Supplementary figures. Fig. S1 Nicotine content of control (Ctrl) and *NtMYC2a*-OE plants before and after topping. Fig. S2 Nicotine contents of in-plate and field cultured *nic* mutants. Fig. S3 Relative expression of nicotine biosynthetic genes in Ctrl (empty vector transformed), *NtMYC2a*-OE and *NtMYC2a*-RI plants. Fig. S4 Relative expression of nicotine biosynthetic genes in plants under N-normal condition. Fig. S5 Schematic diagram of the nicotine biosynthetic pathway. Fig. S6 Representative phenotype of in-plate-cultured tobacco plants under N-normal and N-rich conditions. Fig. S7 Fold change of nitrogen-enhanced expression of nicotine biosynthetic genes. Fig. S8 Analysis of G-boxes in the promoters of *NtODC* (a) and *NtADC* (b) homologues. SI-3 Supplementary tables. Table S1 Primers used for gene cloning and qRT-PCR.

## Data Availability

The datasets and materials that support the findings of this study can be obtained by contacting the corresponding author. The original data in this study are included in the paper and its supplementary materials.

## References

[CR1] Bian S, Sui X, Wang J, Tian T, Wang C, Zhao X, et al. NtMYB305a binds to the jasmonate-responsive GAG region of NtPMT1a promoter to regulate nicotine biosynthesis. Plant Physiol. 2022;188(1):151–66. 10.1093/plphys/kiab458. 34601578 PMC8774768

[CR2] Hou X, Singh SK, Werkman JR, Liu Y, Yuan Q, Wu X, et al. Partial desensitization of MYC2 transcription factor alters the interaction with jasmonate signaling components and affects specialized metabolism. Int J Biol Macromol. 2023;252:126472. 10.1016/j.ijbiomac.2023.126472. 37625752

[CR3] Shi R, Allen Z, Steede T, Lewis RS. Field performance, alkaloid quantities, and transcriptome profiles of nearly isogenic tobacco lines possessing alternative allelic combinations at the *Nic1*, *Nic2*, and *Myc2a* loci. BMC Genomics. 2025;26(1):566. 10.1186/s12864-025-11764-x. 40481397 PMC12142837

[CR4] Shoji T, Hashimoto T, Saito K. Genetic regulation and manipulation of nicotine biosynthesis in tobacco: strategies to eliminate addictive alkaloids. J Exp Bot. 2024;75(6):1741–53. 10.1093/jxb/erad341. 37647764 PMC10938045

[CR5] Sui X, He X, Song Z, Gao Y, Zhao L, Jiao F, et al. The gene NtMYC2a acts as a “master switch” in the regulation of JA-induced nicotine accumulation in tobacco. Plant Biol Stuttg. 2021;23(2):317–26. 10.1111/plb.13223. 33236500

[CR6] Wang S, Shi Q, Li W, Niu J, Li C, Zhang F. Nicotine concentration in leaves of flue-cured tobacco plants as affected by removal of the shoot apex and lateral buds. J Integr Plant Biol. 2008;50(8):958–64. 10.1111/j.1744-7909.2008.00684x. 18713345

[CR7] Wari D, Aboshi T, Shinya T, Galis I. Integrated view of plant metabolic defense with particular focus on chewing herbivores. J Integr Plant Biol. 2021;64(2):449–75. 10.1111/jipb.13204. 34914192

[CR8] Weidner M, Martins R, Müller A, Simon J, Schmitz H. Uptake, transport and accumulation of nicotine by the Golden Potho (*Epipremnum aureum*): the central role of root pressure. J Plant Physiol. 2005;162(2):139–50. 10.1016/j.jplph.2004.07.012. 15779824

[CR9] Zhang HB, Bokowiec MT, Rushton PJ, Han SC, Timko MP. Tobacco transcription factors NtMYC2a and NtMYC2b form nuclear complexes with the NtJAZ1 repressor and regulate multiple jasmonate-inducible steps in nicotine biosynthesis. Mol Plant. 2012;5(1):73–84. 10.1093/mp/ssr056. 21746701

