# Supplementary material for: A negative feedback cascade controlling jasmonate-induced nicotine biosynthesis revealed by an in-plate nicotine assay
Source: Mol Hortic. 2026 Sep 8;6:76. doi: 10.1186/s43897-026-00249-4 (PMC13551806; doi:10.1186/s43897-026-00249-4)
Supplement: Supplementary file 1 [file 43897_2026_249_MOESM1_ESM.docx]

**A negative feedback cascade controlling jasmonate-induced nicotine biosynthesis revealed by an in-plate nicotine assay**

Qing Xu^1^, Ke Xu^1^, Haihui Cao^1^, Yulong Gao^2^, Peng Li^3^, Wenjing Wang^1^, Yong Chen^3^, Michael P. Timko^4^, Zhongfeng Zhang^1^, Bingwu Wang^2,^*, and Hongbo Zhang^1,^*

^1^Key Laboratory of Synthetic Biology of Ministry of Agriculture and Rural Affairs, Tobacco Research Institute, Chinese Academy of Agricultural Sciences, Qingdao 266101, China;

^2^Yunnan Academy of Tobacco Agricultural Sciences, Kunming 650021, China;

^3^Beijing Life Science Academy (BLSA), Beijing 102209, China;

^4^Department of Biology, University of Virginia, Charlottesville, Virginia 22904, USA.

**Author for correspondence:**

Hongbo Zhang, PhD

Tobacco Research Institute, Chinese Academy of Agricultural Sciences, Qingdao 266101, China

Email: [zhanghongbo@caas.cn](mailto:zhanghongbo@caas.cn)

ORCID: 0000-0002-6072-9747

Bingwu Wang, PhD

Yunnan Academy of Tobacco Agricultural Sciences, Kunming 650021, China

Email: [bwwang76@hotmail.com](mailto:bwwang76@hotmail.com)

ORCID: 0000-0002-2234-503X

**SI-1 Supplementary materials and methods**

### **Plant materials and growing conditions**

Tobacco (Nicotiana tabacum L. cv TN90) was used for gene expression assays and transgenic plant development. Plants expressing HA-tagged NtMYC2a (NtMYC2a-OE) were generated as described previously. To construct NtODC1 and NtADC1 overexpression binary vectors, coding sequences of NtODC1 (GenBank Accession: NW_015934162) and NtADC1 (GenBank Accession: NM_001325624) were amplified and cloned into the pENTR-D-TOPO vector (Invitrogen, Carlsbad, USA). The resulting constructs were subsequently introduced into the binary vector pMDC-attR-Flag via Gateway recombination, yielding pMDC-NtODC1-Flag and pMDC-NtADC1-Flag. These vectors were introduced into TN90 tobacco via Agrobacterium-mediated transformation (Bian *et al*., 2022) to generate plants overexpressing NtODC1-Flag (NtODC1-OE) and NtADC1-Flag (NtADC1-OE), respectively. These lines were crossed with NtMYC2a-OE plants to obtain hybrids co-expressing NtMYC2a with either NtODC1 or NtADC1. Primers used for cloning are listed in Table S1. Tobacco plants transformed with empty vector were used as control.

For the in-plate nicotine production assay (IPNPA), tobacco seeds were surface-sterilized with 5% bleach containing 0.1% Tween 20, germinated on MS medium containing Kanamycin (for NtMYC2a-OE plants), Hygromycin (for NtODC1/NtADC1-OE plants), or both antibiotics (for hybrids) at 23 °C under a 14-h-light/10-h-dark photoperiod for 14 days. Then, the antibiotic resistant plants were transferred onto MS medium plates containing 60 mM N (N-normal) or 120 mM N (N-rich), supplemented with 0 µM (mock) or 3 µM MeJA (treatment), and cultured for an additional 15 days. NH₄NO₃ was selected for N supplementation for it is a common inorganic nitrogen for MS medium and provides both ammonium (NH₄⁺) and nitrate (NO₃⁻). Tissues above the cotyledons (including cotyledons and aerial parts) were collected for nicotine measurement, while tissues below the cotyledons (hypocotyl and roots) were harvested for gene expression analysis at designated time points. For topping treatment, tobacco plants were pot-grown for 16 weeks until flowering. Middle internodal leaves were collected before and after topping for nicotine extraction and quantification.

### **Nicotine measurement**

Nicotine content was measured as described previously with minor modifications (Zhang *et al.*, 2012). Briefly, dried leaf samples were ground to a fine powder, and 0.1 g was alkalized with 1 mL of 10% (w/v) NaOH and extracted with 5 mL ethyl acetate for 2 min with shaking. Nicotine was quantified using an Agilent 7890A gas chromatograph equipped with a DB-5MS column. A nicotine standard (Sigma-Aldrich) was used for calibration.

### **RNA extraction and qRT-PCR**

Total RNA was extracted using TRIzol reagent (Invitrogen, Carlsbad, USA) and reverse-transcribed into cDNA using the PrimeScript First-Strand cDNA Synthesis Kit (TaKaRa, Japan). qRT-PCR was performed on a QuantStudio 5 Real-Time PCR System (Thermo Fisher Scientific, Waltham, USA) with ChamQ Universal SYBR qPCR Master Mix (Vazyme, Nanjing, China). Primers were designed from conserved regions of target genes (Table S1). NtActin was used as the internal reference (Zhang *et al.*, 2012). Five biological replicates were analyzed for each gene.

### **Electrophoretic mobility shift assay**

The coding sequence of NtMYC2a was amplified and cloned into the pET28b vector (Novagen, Madison, USA) via *Nco* I and *Xho* I sites for expression of His-tagged protein in E. coli BL21. DIG-labeled double-stranded probes containing G-box elements from the NtODC1 and NtADC1 promoters were synthesized by Sangon Biotech (Shanghai, China). EMSA was performed as described (Bian *et al*., 2022), and signals were detected using the DIG High Prime DNA Labeling and Detection Starter Kit (Roche). Probe sequences are listed in Table S1.

### **Transactivation assay**

Transactivation assays were performed as described (Bian *et al*., 2022). Truncated promoter fragments of NtODC1 (–1374, –990, and –850 bp) and NtADC1 (–1994, –1550, –1341, and –1042 bp) were cloned into the pBT10-GUS vector and introduced into A. tumefaciens LBA4404. Twenty-day-old control and NtMYC2a-OE seedlings were infected with reporter-containing *Agrobacteria* and co-cultivated on MS medium with 100 µM acetosyringone for 2 days in darkness, then transferred to medium containing 250 mg/L cefotaxime for 3 days. GUS staining and activity assays were performed as described (Bian *et al*., 2022). GUS activity was quantified using a 4-methylumbelliferone (MU) standard and expressed as pmol MU/min/mg protein. Primers for promoter amplification are listed in Table S1.

### **ChIP assay**

ChIP (chromatin immunoprecipitation) was performed as described (Bian *et al*., 2022). Briefly, 1 g of leaves from 4-week-old seedlings was cross-linked in 20 mL 1×PBS buffer containing 1% formaldehyde (v/v) for 15 min under vacuum, quenched with glycine at concentration of 0.125 M, and ground into a powder in liquid nitrogen after three time washing with 1×PBS buffer. Chromatin was sheared by ultrasonication sonicated and immunoprecipitated with anti-HA antibody (Roche, Mannheim, Germany) and ssDNA/protein G agarose. Precipitated DNA was quantified by qPCR with primers flanking G-box regions, and NtActin was used as the internal control. Primers are listed in Table S1.

### **Statistical analysis**

Statistical analysis was performed using Microsoft Excel based on the data of five biological replicates for each assay. Statistically significant differences were assessed by one- or two-way ANOVA followed by Tukey’s test or Student’s *t*-test as indicated. Data are presented as mean ± SD.

**SI-2 Supplementary figures**

**Figure S1** Nicotine content of control (Ctrl) and *NtMYC2a*-OE plants before and after topping. Plants were cultured with 75kg N per acre. The topping-induced nicotine increase is indicated by percentage. Presented data for each genotype are from three independent lines. Lowercase letters indicate significant differences (Tukey's test, *P* < 0.05).

**Figure S2** Nicotine contents of in-plate and field cultured *nic* mutants. (**a**) In-plate cultured *nic* mutants without (Mock) or with MeJA treatment. Bar represents 0.5 cm. (**b**) Nicotine contents of in-plate cultured *nic* mutants without (Mock) or with MeJA treatment. (**c**) Nicotine contents of field cultured *nic* mutants with 75kg N per acre. (**d**) Nicotine contents of field cultured *nic* mutants with 150kg N per acre. Field experiments were performed at Qingdao, China. NC95, wild type; MAFC5, *Nic1nic2*; MLAF34, *nic1Nic2*; LAFC53, *nic1nic2*. Lowercase letters indicate significant differences (Tukey's test, *P* < 0.05).

**Figure S3** Relative expression of nicotine biosynthetic genes in Ctrl (empty vector transformed), NtMYC2a-OE and *NtMYC2a*-RI plants. Expression in Ctrl plants without MeJA was set to 1. −MeJA, untreated; +MeJA, MeJA-treated. Presented data for each genotype are from three independent lines. Lowercase letters indicate significant differences (Tukey’s test, P < 0.05).

**Figure S4** Relative expression of nicotine biosynthetic genes in plants under N-normal condition. Expression in Ctrl plants without MeJA was set to 1. −MeJA, untreated; +MeJA, MeJA-treated. Presented data for each genotype are from three independent lines. Lowercase letters indicate significant differences (Tukey’s test, P < 0.05).

**Figure S5** Schematic diagram of the nicotine biosynthetic pathway. Key enzymes of the pathway are highlighted in blue color. NtODC, ornithine decarboxylase; NtADC, arginine decarboxylase; NtPMT, putrescine methyltransferase; NtQPT, quinolinic acid phosphoribosyltransferase; NtBBL, berberine bridge enzyme-like; NtA622, PIP-family oxidoreductase.

**Figure S6** Representative phenotype of in-plate-cultured tobacco plants under N-normal and N-rich conditions. Ctrl, empty vector transformed. Areas in red rectangles are shown in Figure 1e with magnification. Bar = 0.5 cm.

**Figure S7** Fold change of nitrogen-enhanced expression of nicotine biosynthetic genes. Values are calculated by comparing the relative gene expression in plants under N-rich condition with that under N-normal condition. −MeJA, untreated; +MeJA, MeJA-treated. Presented data for each genotype are from three independent lines. Lowercase letters indicate significant differences (Tukey’s test, P < 0.05).

**Figure S8** Analysis of G-boxes in the promoters of *NtODC* (**a**) and *NtADC* (**b**) homologues. G-boxes (gray colored) and the flanking sequences are shown, and the positions of G-boxes in promoters are indicated. Gene accessions at NCBI GenBank are as followings: *NtODC1* (Gene ID: 107801491), *NtODC2* (LOC107815922), *NtADC1* (LOC107796783), *NtADC1A* (Gene ID: 107774919), and *NtADC2* (LOC107767963).

**SI-3 Supplementary tables**

**Table S1** Primers used for gene cloning and qRT-PCR.

| **Primers for gene cloning** | |
| --- | --- |
| *NtODC1* | AAAGGCGCGCCATGGCCGGCCAAACAA  AAAGCGGCCGCGCTTGGATAAGCATA |
| *NtADC1* | AAAGGCGCGCCATGCCGGCCCTAGGTT  AAACCGCGGAGCAGTGCAATAGGACC |
| *NtMYC2a* | TAAGAAGGAGATATACCATGGATGACTGATTACAGCTTACCCACCA  GTGGTGGTGGTGGTGCTCGAGGCGTGTTTCAGCAACTCTGGA |
| **Primers for qRT-PCR** | |
| *NtODC* | ACCCGAAATCCGAACTCTTGCTC  CCGCTGCCGATGTGGAAGG |
| *NtADC* | CTCGGCTATGCCTTCTCTCG  AGATCCGGAGACCCAATGGA |
| *NtPMT* | TGGGAAGGTTCTGACTTTGGATGG  ACCAATTCCTCCGCCGATGATC |
| *NtQPT* | AAGCCACCAGCACACCCAAC  TCGTCGCCTTACAAGTCACATCTC |
| *NtBBL* | TGCTGCTGATAATGTCGTTGATGC  CCGCCGCCACCTCTGATTG |
| *NtA662* | GCTGCATCACTCTTCGCTCTAAAG  TTTGTCTCTAGGAGGGGTTTTGGC |
| *NtActin* | CCACACAGGTGTGATGGTTG  GTGGCTAACACCATCACCAG |
| **Nucleotide sequences of EMSA probes (One strand shown for each, bases of complementary replacement are underlined)** | |
| *PrODC1*-G1 | CAAATCTAGGATTCTATATATGCACATGTCGAAACTTTAAATATTTAAT |
| *PrODC1*-G2 | TTCCCTAAGATCCACATTTTTCATGTGCTCTTTTCTTTCTTTATCCCG |
| *PrODC1*-G3 | GGCTAAGCAAAACAAATCATGCACTTGCATCTCTATTTTAGTATGAGT |
| *PrODC1*-mG1 | CAAATCTAGGATTCTATATATGATAGCCTCGAAACTTTAAATATTTAAT |
| *PrODC1*-mG2 | TTCCCTAAGATCCACATTTTTTCGTGACTCTTTTCTTTCTTTATCCCG |
| *PrODC1*-mG3 | GGCTAAGCAAAACAAATCATGTCAGCTCATCTCTATTTTAGTATGAGT |
| *PrADC1*-G1 | AAAACTAATTAATTAATCTATCATGTGATCCCCACTATGTATTTTTAA |
| *PrADC1*-G2 | CACTATATTAAGAGTGGTTATCATTTGTAATGGAGAGACATACACACA |
| *PrADC1*-G3 | TACATAGCACAATAGCACCCTCAATTGCAAAAGTCCAAAGCCAAGGGT |
| *PrADC1*-G4 | TGTTTATACTCAATTTTTTTGCAAATGTGTCTAAAATGATAGGACTTG |
| *PrADC1*-mG1 | AAAACTAATTAATTAATCTATTCGTGAATCCCCACTATGTATTTTTAA |
| *PrADC1*-mG2 | CACTATATTAAGAGTGGTTATTTACGTTAATGGAGAGACATACACACA |
| *PrADC1*-mG3 | TACATAGCACAATAGCACCCTATCTGACAAAAGTCCAAAGCCAAGGGT |
| *PrADC1*-mG4 | TGTTTATACTCAATTTTTTTGACGTAATGTCTAAAATGATAGGACTTG |
| **Primers for GUS** | |
| *NtODC1*-P1-F | TCCCCGCGGGGATCCTGACGCAGTATGGGTGTGTAT |
| *NtODC1*-P2-F | TCCCCGCGGGGAGGTATATGCTTTGTTCTGAGATGC |
| *NtODC1*-P3-F | TCCCCGCGGGGACCGAGATTTGAAAATAAAATAAAT |
| *NtODC1*-R | CCAAACAAAGGAAGAAAACAGAGA |
| *NtADC1*-P1-F | TCCCCGCGGGGACGGAAATTCATATCGTTTCTC |
| *NtADC1*-P2-F | TCCCCGCGGGGACTCAAAATGGGATTCCCCCAC |
| *NtADC1*-P3-F | TCCCCGCGGGGAGGGTTGCATTCATTTCTTTTA |
| *NtADC1*-P4-F | TCCCCGCGGGGACCTCGCATAGATTTACCTTCT |
| *NtADC1*-R | CTCTTCTTCTTCTGTTAAAGAATC |
| **Primers for ChIP-qPCR** | |
| *NtODC1*-G1 | CGCGGCCATAAATATGTTAGCA |
|  | TCGTCGTTTTACAATAAACTCAAGT |
| *NtODC1*-G2 | AGTTGGCTGTCCTTTCCCTA |
|  | GCTTAGCCAGCCTCTTTCTCT |
| *NtODC1*-G3 | TTAGAGAAAGAGGCTGGCTAAG |
|  | TCTCTGATTAAAGGCGAGAACATTT |
| *NtADC1*-G1+G2 | TGCGGAAATCACGACTTGAA |
|  | TGAATGTGTGTATGTCTCTCCAT |
| *NtADC1*-G3 | ACCACGTATTTTTAGAACTGCGT |
|  | GGAACCCTTGGCTTTGGACT |
| *NtADC1*-G4 | ACTGAAGAACAATAGCCTCGCA |
|  | TTTGCAAGTCCTATCATTTTAGACA |
| *NtActin* | CCACACAGGTGTGATGGTTG |
|  | GTGGCTAACACCATCACCAG |
